# AIFM-ed Curriculum Framework for Postgraduate Family Medicine Education on Artificial Intelligence: Mixed Methods Study

**DOI:** 10.2196/66828

**Published:** 2025-04-25

**Authors:** Raymond Tolentino, Fanny Hersson-Edery, Mark Yaffe, Samira Abbasgholizadeh-Rahimi

**Affiliations:** 1 Department of Family Medicine Faculty of Medicine and Health Sciences McGill University Montreal, QC Canada; 2 Department of Family Medicine St. Mary’s Hospital Center Integrated University Centre for Health and Social Services of West Island of Montreal Montreal, QC Canada; 3 Mila-Quebec Montreal, QC Canada; 4 Lady Davis Institute for Medical Research Jewish General Hospital Montreal, QC Canada; 5 Faculty of Dental Medicine and Oral Health Sciences McGill University Montreal, QC Canada

**Keywords:** artificial intelligence, family medicine, curriculum, framework, postgraduate education

## Abstract

**Background:**

As health care moves to a more digital environment, there is a growing need to train future family doctors on the clinical uses of artificial intelligence (AI). However, family medicine training in AI has often been inconsistent or lacking.

**Objective:**

The aim of the study is to develop a curriculum framework for family medicine postgraduate education on AI called “Artificial Intelligence Training in Postgraduate Family Medicine Education” (AIFM-ed).

**Methods:**

First, we conducted a comprehensive scoping review on existing AI education frameworks guided by the methodological framework developed by Arksey and O’Malley and Joanna Briggs Institute methodological framework for scoping reviews. We adhered to the PRISMA-ScR (Preferred Reporting Items for Systematic Reviews and Meta-Analyses extension for Scoping Reviews) checklist for reporting the results. Next, 2 national expert panels were conducted. Panelists included family medicine educators and residents knowledgeable in AI from family medicine residency programs across Canada. Participants were purposively sampled, and panels were held via Zoom, recorded, and transcribed. Data were analyzed using content analysis. We followed the Standards for Reporting Qualitative Research for panels.

**Results:**

An integration of the scoping review results and 2 panel discussions of 14 participants led to the development of the AIFM-ed curriculum framework for AI training in postgraduate family medicine education with five key elements: (1) need and purpose of the curriculum, (2) learning objectives, (3) curriculum content, (4) organization of curriculum content, and (5) implementation aspects of the curriculum.

**Conclusions:**

Using the results of this study, we developed the AIFM-ed curriculum framework for AI training in postgraduate family medicine education. This framework serves as a structured guide for integrating AI competencies into medical education, ensuring that future family physicians are equipped with the necessary skills to use AI effectively in their clinical practice. Future research should focus on the validation and implementation of the AIFM-ed framework within family medicine education. Institutions also are encouraged to consider adapting the AIFM-ed framework within their own programs, tailoring it to meet the specific needs of their trainees and health care environments.

## Introduction

The College of Family Physicians of Canada (CFPC) establishes standards for postgraduate family medicine training and its accreditation [1]. It promotes a competency-based curriculum model known as Triple C (comprehensive, continuous, and centered in family medicine) [2] based on the Canadian Medical Education Directives for Specialists (CanMEDS)—Family Medicine framework [3] and on assessment objectives for certification in family medicine [4]. To ensure that medical curricula respond to new developments in health care, education, and societal trends, they must undergo periodic review, modification, and renewal [5-9]. Accordingly, a number of new content areas have been introduced in the recent past into the family medicine curricula. They include leadership [10], social determinants of health [11], ethics [12], global health [13-15], and physician wellness and burnout [16-18]. The increasing complexity of the medical needs of an aging population, the exponential growth in medical knowledge, and an increasingly digitalized environment suggest the need for digital-mediated solutions to support medical practitioners.

Artificial intelligence (AI) and its applications have made a rapid impact on many segments of society, including medicine [19] and notably, in primary health care [20]. While there is no universal consensus on the definition of AI, the World Health Organization [21] describes it as “the performance by computer programs of tasks that are commonly associated with intelligent beings.” The introduction, integration, and implementation of AI-based tools and systems into family medicine education and practice assume an adequately trained cohort of users, but to date, training of family physicians on relevant aspects of AI to ensure effective and safe implementation has been absent or inconsistent [20,22]. As such, the CFPC’s Outcomes of Training project has identified digital care and health informatics as a training gap and an area for educational enhancement requiring priority attention across the 17 family medicine postgraduate programs in Canada [23,24]. There have been efforts to include AI education globally within each level of medical training. These efforts are led by national medical associations such as the UK National Health Service, the US American Medical Association, and Canada’s Royal College of Physicians and Surgeons. They have released documents recommending policies for integrating AI within their respective medical educational institutions [25-27].

Initiatives of AI teaching directed at physicians already in practice include the development of a continuing professional development 3-module CFPC Learn e-course titled, “Artificial Intelligence for Family Medicine” [28]. The first module of this course reviews the basic functionality of AI with applications in family medicine, while the second module focuses on core terminology and related concepts as well as potential harms or risks associated with AI. The last module reviews the concepts of the first 2 and focuses on learning how to tell if an AI-based tool is working well [28].

Competency about a particular subject has been described as the ability to carry out a certain task or action at a basic or acceptable level [29]. Liaw et al [30] have recently proposed six competency domains for family medicine training in AI: (1) foundational knowledge (What is this tool?), (2) critical appraisal (Should I use this tool?), (3) medical decision-making (When should I use this tool?), (4) technical use (How do I use this tool?), (5) patient communication (How should I communicate with patients regarding the use of this tool?), and (6) awareness of unintended consequences (What are the “side effects” of this tool?).” These authors suggest that such competencies can be integrated within current residency training during existing sessions on health informatics or evidence-based medicine but emphasize that these competencies are a “point of departure” and must be further worked on [30].

A curriculum framework can be described as “a core policy document that describes a range of requirements, regulations and advice which should be respected by all stakeholders in the education system, and which should guide the work of schools, teachers and the developers of other curriculum documents” [31]. Curriculum frameworks allow for a visual and detailed roadmap to develop and implement a curriculum [32]. Input from an interdisciplinary team of medical educators, AI experts, end users, researchers, and curriculum designers [33] can effectively support the development of a curriculum framework for teaching AI in family medicine postgraduate training programs. Our comprehensive review of the available curriculum frameworks [34,35] highlighted that there is no framework designed specifically for family medicine residency and no paper that described a systematic approach to design one. From the 2 frameworks uncovered, one framework was incomplete, while the other framework was brief and focused on ophthalmology [34]. The ophthalmology curriculum framework lacks adaptability, as it may prove inadequate for family medicine residency due to the diverse, community-based nature of family medicine, which differs significantly from the highly technological and hospital-based focus of ophthalmology.

Considering the gaps mentioned previously and the foundational importance of curriculum frameworks in the creation of new educational structures, our objective was to design and develop a curriculum framework for AI family medicine education, that is, Artificial Intelligence Training in Postgraduate Family Medicine Education (AIFM-ed), ensuring alignment with current competencies and educational goals. To achieve this, a combination of validated methods including 2 national expert panel discussions were conducted, supplemented by a previous comprehensive systematic scoping review [34]. Developing a framework based on expert insights would help address gaps in AI education and provide an adaptable guide for family medicine educators, curriculum designers, postgraduate residency program directors, medical education researchers, and policy makers in health care education. Due to the systematic approach in designing this framework, audiences can adopt this framework to other fields and specialties, considering that our review did not find any systematically developed frameworks.

## Methods

### Study Design

For the construction of an AIFM-ed framework, we followed the analysis, design, development, implementation, and evaluation model for instructional design, using the first 3 activities to guide our work. We followed a two-step approach suggested by Redwood-Campbell et al [36] for framework development, wherein (1) a review of the literature was made focusing on curriculum frameworks and core competencies for AI education in medicine [34,35] and (2) a working group used qualitative or consensus methods for final development of the framework.

Our scoping review aimed to synthesize knowledge from the literature on curriculum frameworks and current educational programs that focus on the teaching and learning of AI for medical students, residents, and practicing physicians, and adhered to PRISMA-ScR (Preferred Reporting Items for Systematic Reviews and Meta-Analyses extension for Scoping Reviews) guidelines. Details of this comprehensive study have been published elsewhere [34,35]. Our review specifically identified several AI educational curricula programs (eg, courses, workshops, webinars, and projects) and 2 curriculum frameworks for AI education, one outlining a broad framework for any level of education [37], while the other described a complete framework for ophthalmology residency education [38].

The outcome of our review was the identification of early concepts that could be applied to elements of the curriculum framework for family medicine and AI [34,35]. This initial curriculum framework was later used during the panel discussion as part of the co-development and redesigning of the framework. This discussion applied the curriculum framework structure described by Obadeji [39], which examines six common elements: (1) the need and the purpose of a curriculum or a program, (2) learning objectives and outcomes, (3) course content that will facilitate the accomplishment of the objectives or learning outcomes, (4) organization of the content, (5) implementation of curriculum, and (6) curriculum evaluation and refinement. This study examined all elements except the final element (curriculum evaluation and refinement). The initial framework was deemed successful by the expert team based on the following indicators: relevance to medical educators and curriculum designers, alignment to current family medicine competencies and educational goals, clarity of AI-specific content, and its potential for further validation. However, we acknowledge that further studies are needed.

### Qualitative Methodology

The expert panel methodology follows the SRQR (Standards for Reporting Qualitative Research) checklist [40]. Expert panels help to attempt to reach consensus on controversial subjects [41,42] such as the risk of AI tools leading to reduced proficiency in independent critical thinking and clinical judgment among physicians. The use of qualitative consensus methods for curriculum development facilitates input from a wide range of stakeholders (eg, physicians and curriculum developers) in order to assess and validate expert knowledge [43]. The use of expert panel discussions to assist in creating curricula has become established in pedagogical research and development [44]. Examples within the field of medicine include discussions around social determinants of health for undergraduate medical education [45], telemedicine opportunities for postgraduate medical education [46], and geriatric oncology in continuing medical education [47].

### Participant Recruitment and Sampling Strategy

Our panel size fell within the recommended average of 8 members or a median of 6 [48]. The definition of an expert in our case is flexible due to the limited knowledge and experience on this emerging topic; this is emphasized by Duncan et al [49], who state that, “[t]oo narrow a definition, however, can restrict the number of potential participants.” In our case, we chose experts according to the definitions of Fink et al [41], which state that they must be, “representative of their professional group, with either sufficient expertise not to be disputed or the power required to instigate the findings.” This was reinforced by Mead and Mosely [50], which state that, “experts can be defined in a number of ways, such as their position in a hierarchy [...] or as recommended by other participants in a study.” Therefore, from these definitions, we selected panelists based on their academic qualifications, their number and relevance of AI-related publications, professional experience within the development, implementation or research of AI, and finally, any participation in AI-specific projects or conferences.

For panel 1, we reached out to family medicine (clinical) educators from affiliated universities and professional organizations across Canada via email. Snowballing by this initial group generated the names of others known as family medicine educators. For panel 2, family medicine residents were invited from an initial group of residents who were knowledgeable and aware of AI, and that initial group helped to recruit relevant residents for this study.

Each participant voluntarily participated in the study by providing their explicit consent and agreement, which was confirmed through email correspondence. To uphold confidentiality, data were safeguarded through limited, secure data access, the disposal of audiotapes after transcription, and the anonymous analysis of transcripts.

### Ethical Considerations

This study involved a panel discussion with experts, which does not require formal ethics board approval under the Economic and Social Research Council Framework for Research Ethics guidelines [51]. According to these guidelines as well as Canada’s Tri-Council Policy Statement on Ethical Conduct for Research Involving Humans, research that presents minimal risk and does not involve sensitive information may be exempt from formal ethics review [52]. This study adhered to these recognized guidelines, ensuring that all participants were treated in accordance with principles of research integrity, voluntary participation, and informed consent.

### Participant Eligibility Criteria

Input from 2 different types of panelists was desired, and they were included as participants within 2 distinct expert panels. The first included family medicine educators practicing in Canada who were somewhat knowledgeable or have expertise in AI education. No limitations were placed on years of practice experience, years of knowledge or experience in AI, language proficiency, work setting, or the types of patients for whom they provided care. The second expert panel included participants who were at the time of the study family medicine residents at McGill University, and who were somewhat knowledgeable in AI. No limitations were placed on language proficiency, years of knowledge or experience in AI, work settings, or the types of patients they provided care for.

### Data Collection

We conducted a recorded session of each expert panel via Zoom (version 5.16.10; Zoom Video Communications). The use of a web-based expert panel minimizes costs associated with travel; it also mitigates potential biases linked to panelists [53]. Each web-based expert panel discussion was approximately 2 hours long, followed the same format, used congruent discussion guides, and was facilitated by 2 members of the research team (RT and SAR). The discussions began with a brief presentation given by RT on the results of the first step of the project, that is, the comprehensive scoping review in the field [34,35]. Following the presentation, each of the five elements of the curriculum framework: (1) the need and the purpose of a curriculum or a program, (2) learning objectives and outcomes, (3) course content that will facilitate the accomplishment of the objectives or learning outcomes, (4) organization of the content, and (5) implementation of the curriculum were discussed sequentially and at length. When presenting each element, participants were invited to respond and discuss their opinions and thoughts related to each element, allowing for the co-development and redesigning of the framework together.

### Data Analysis

Expert panel discussion data were analyzed using content analysis strategies [54,55] as previously used in a study developed for a training model for nurses using a literature review and expert panel discussions, in which data were analyzed using a descriptive qualitative approach that includes content analysis [56]. In our work, the preparation phase included transcribing the data, immersing in the data, and obtaining a sense of whole through reading the transcript multiple times. In our study, once the recordings from the expert panel discussions were received, one of the authors (RT) listened to the entire recording and subsequently transcribed it verbatim. The next stage of data analysis was the organizing phase, in which open coding and the creating of categories were conducted along with the grouping of codes under higher-order headings. These were carried out by one of the authors (RT) and verified by the senior author (SAR).

As the analysis of data used an inductive approach, no prior coding systems were used, such that coded categories were derived directly from the data [55]. Sentences and phrases from the panelists were captured. In vivo coding was used to prioritize participants’ language and perspectives, while descriptive coding aided in categorizing key themes. Two independent coders reviewed the data (RT and SAR), with discrepancies resolved through discussion between coders and the research team. Saturation was achieved when no new themes emerged during the coding of the final transcript. The final step included the presentation of the final curriculum framework, which resulted from the incidence of codes and categories and its relation to the literature. Codes and categories derived were prioritized and highlighted with how frequently they appeared during the panel discussion as well as the overlap between both groups. These highlighted findings were then compared with existing literature to either support or challenge them. If these codes and categories were supported by the literature, they were subsequently integrated into the framework.

## Results

### Panelists Characteristics

A total of 37 educator and resident experts were invited, 14 for the educator group and 23 for the resident group. Ultimately, 8 from the former and 6 from the latter group participated, for a total of 14 participants. Scheduling problems were the most common reasons for nonparticipation. The characteristics of those included in the expert panel discussion are displayed in Table 1.

**Table 1:** Characteristics of expert panel participants included.

		Educator experts (n=8), n (%)	Resident experts (n=6), n (%)
**Sex**			
	Male	3 (38)	4 (66)
	Female	5 (62)	2 (33)
**Educational background**			
	Doctoral (PhD)	7 (88)	0 (0)
	Master	1 (22)	2 (33)
	Bachelor or MD only	0 (0)	4 (66)
**Affiliation**			
	McGill University	5 (62)	6 (100)
	Other academic institution	3 (38)	0 (0)

### Curriculum Framework for AIFM-ed

#### Overview

**Figure 1:**
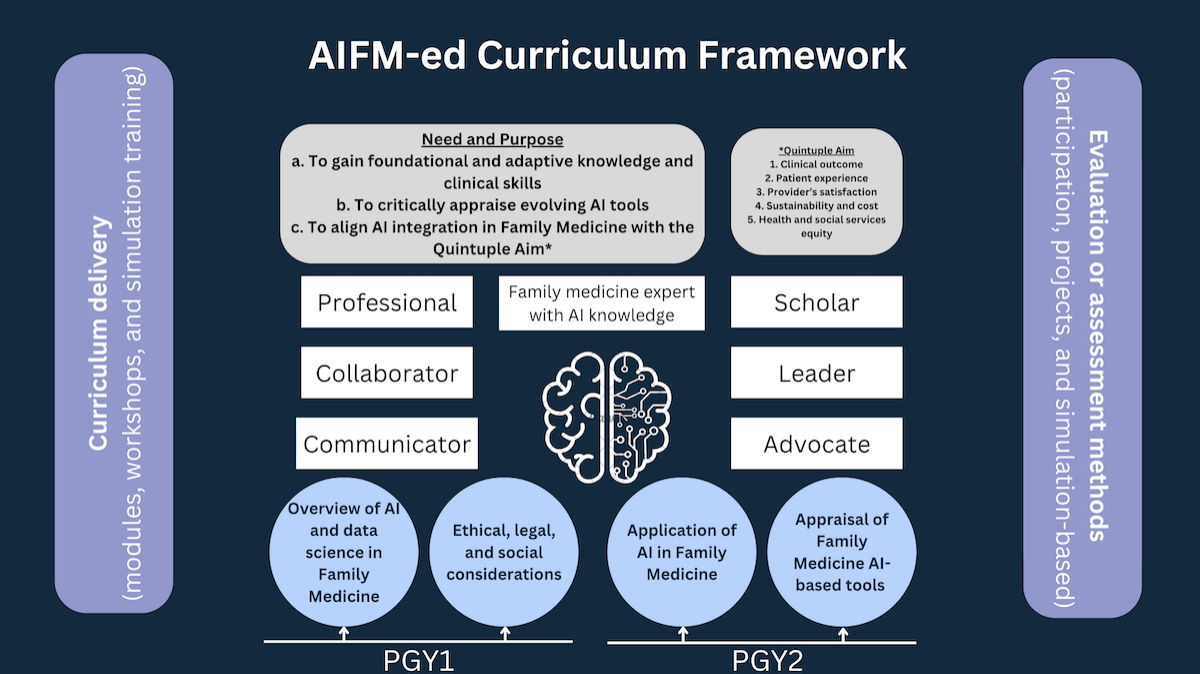
Representation of the AIFM-ed curriculum framework. AI: artificial intelligence; AIFM-ed: Artificial Intelligence Training in Postgraduate Family Medicine Education; PGY: postgraduate year. * refers to the Quintuple Aim.

Our project has identified five elements of the curriculum framework for AI training in postgraduate family medicine education: (1) need and purpose of the curriculum, (2) learning objectives, (3) curriculum content, (4) organization of curriculum content, and (5) implementation of the curriculum. A condensed visual representation of the AIFM-ed curriculum framework is displayed in Figure 1, while each element is discussed in detail below.

#### Element 1: Need and Purpose of the AIFM-ed Curriculum

When modifying a curriculum in family medicine postgraduate training, it is important to understand why it must be changed and for what purpose. Both panels discussed the current low priority of AI curricula. Residents emphasized a lack of exposure in training and practice. Both panels agreed that the integration of an AI curriculum will inevitably become imperative, recognizing its potential as an essential toolset in practice. One educator summarized this thought by saying, “AI will continue to evolve quickly, so a curriculum must be built for the future.”

To describe the need and purpose for AI education in family medicine, we co-developed the following: “The purpose of an AI curriculum for family medicine residents is for future family physicians to: (a) gain foundational and adaptive knowledge and clinical skills, (b) to critically appraise evolving AI tools, and (c) to align AI integration in Family Medicine with the quintuple aim for health care improvement (i.e., improving population health, improving the provider and patient experience, reducing costs, and advancing health equity [57,58]).”

Using various definitions of AI, the educator panel debated what constitutes AI specifically in family medicine. The term “AI-based tools” is used throughout the results of this paper as a way of describing technologies empowered or enabled with AI algorithms to support clinical practice. This term has been used in previous literature on AI in the context of family medicine training [20,30].

#### Element 2: AIFM-ed Learning Objectives

Learning objectives are statements that describe significant and essential learning that learners need to be familiar with and reliably demonstrate at the end of a course or educational program [59]. The following outlines the learning objectives for AI training, aligning with CanMEDS and family medicine roles. Table 2 presents each CanMEDS role on the left column [3], with their affiliated learning objectives for AI family medicine education as structured by participants during the panel on the right column. Although the learning objectives are comprehensive and their practical application for most family doctors may be limited, they are ideal for advancing the knowledge and skills of AI-empowered family physicians.

**Table 2:** Learning objectives discussed during panels about artificial intelligence (AI) in relation to Canadian Medical Education Directives for Specialists (CanMEDS) roles.

CanMEDS roles		The learner engaged in AI education will be able to
**Family medicine expert with AI knowledge**		
	Family physicians are skilled generalists who should be able to understand and use technology including AI tools to provide high-quality, responsive, community-adaptive care across the lifecycle, from prevention to palliation, in multiple settings, and for diverse populations.	Explain a basic understanding of AI and basic concepts in relation to family medicine. Demonstrate the use of AI-based tools for family medicine by showing how to use the tool and understand the output. Critique and decide on when to use an AI-based tool over another health care resource. Recognize AI-based tools’ perceived biases and discriminatory behavior (eg, an AI-based tool diagnosing skin conditions mainly trained on images of lighter skin tones may be less accurate in detecting conditions in individuals with darker skin tones) and the results demonstrated by AI-based tools where the learner will be able to solve and prevent further effects.
**Communicator**		
	Family physicians foster life-long therapeutic relationships with patients and their families. This incorporates the dynamic exchanges that occur before, during, and after the medical encounter that facilitates gathering and sharing essential information for effective patient-centered health care [3].	Explain to patients the current AI-based tool they are using and its results. Address relevant gaps of understanding of AI tools among patients such as differing cultural perspectives and digital health literacy.
**Collaborator**		
	Family physicians work with patients, families, communities, and other health care providers to provide safe, high-quality, patient-centered care [3].	Practice a collaborative team-based approach, including establishing positive and continuing working relationships with relevant stakeholders in relation to developing, implementing, and improving the quality of AI-based tools.
**Leader**		
	Family physicians must actively contribute to implementing and maintaining a high-quality health care system and take responsibility for delivering excellent patient care. This includes prioritizing and using health care resources efficiently, executing tasks collaboratively with colleagues, and contributing to ongoing quality improvement initiatives within their own practice and its management [3].	Identify which AI-based tools are appropriate for the clinical practice of family physicians. Allocate AI-based tools, when available, to specific tasks (eg, administrative work) in order for optimal patient care and practice management. Analyze incidents of use of AI-based tools, appraise AI-based tools, and resolve any issues to avoid patient injury.
**Advocate**		
	Family physicians leverage AI-driven insights to advocate for patients and communities, using their expertise to identify health needs, drive meaningful change, and mobilize resources for improved care outcomes [3].	Extend AI-based tools and resources, when available and known, with other family physicians and family medicine communities. Advocate for established AI-based tools, when available, to patients with the aim of improving their health outcomes.
**Scholar**		
	Family physicians demonstrate a lifelong commitment to excellence in practice through continuous learning and teaching others; gather, combine, and evaluate evidence; and contribute to the creation and dissemination of knowledge [3].	Participate in scholarly activities related to AI that benefit professional growth, clinical practice, and patients. Maintain or enhance one’s own knowledge and skills through professional educational activities related to AI and ongoing self-directed learning.
**Professional**		
	Family physicians are committed to the health and well-being of their patients and society through competent medical practice; accountability to their patients, the profession, their colleagues, and society; profession-led regulation; ethical behavior; and maintenance of personal well-being [3].	Recognize and appropriately respond to ethical, legal, and social issues encountered in practice, as it relates to AI-based tools and family medicine by communicating to the proper channels and resources (eg, AI and data experts, information technology specialists, ethics boards, and lawyers).

#### Element 3: AIFM-ed Curriculum Content

When developing a curriculum, a crucial task is to identify relevant subject knowledge, skills, attitudes, and behaviors that will help form the learning objectives [39]. Currently, there is no required AI education in Canadian undergraduate medical education. However, both educators and residents in our study agreed that for AI to be efficiently introduced in family medicine residency, it must be preceded by education in undergraduate medical education. This earlier introduction of principles and concepts of AI will facilitate learning the more difficult material that is to come. The panels envisaged a basic stream of education in residency for those who had no exposure in undergraduate years. This would address fundamentals and basic knowledge of AI (eg, history, AI model development process, and core algorithms). A more advanced stream of AI education for residents would summarize the fundamentals and focus on how to use AI-based tools (applications) along with how to decide when to use and evaluate them (critical appraisal).

Residents noted that understanding how AI-based tools are used in clinical practice was the preferred content area for study, with less attention devoted to ethical, legal, and social considerations of AI. A resident put this in context, noting that they “do not need or want to learn the history of ChatGPT, but rather how to write effective prompts within this natural language processing chatbot.” Table 3 summarizes the key concepts and areas of interest that family physicians should learn and content to include in the curriculum, as viewed by the participants.

**Table 3:** The curricular concepts and topics of relevance to family physicians.

Main curricular topic		Subtopics
**Overview of AI^a^ and data science in family medicine**		
	Providing an overview of AI definitions and concepts including machine learning as well as topics related to data science (eg, mathematics and statistics) and clinical epidemiology for family medicine.	Review of AI (definitions and concepts) as it relates to family medicine Introduction to AI and fundamentals of data science in family medicine Strength and limitations of AI-based tools
**Ethics, legal, and social considerations**		
	Understanding the ethical, legal, and social concerns of AI as it impacts family medicine clinical practice.	Ethics, patient rights, data security, and confidentiality Liabilities and regulatory and policy considerations Equity, diversity, and inclusion of AI
**Application of AI in family medicine**		
	Understanding how to choose and engage with AI-based tools in clinical settings and workflows with the ability to understand, interpret, and apply results of AI systems in clinical practice.	Clinical practice management and operation Preventative care and risk profiling (eg, mental health and chronic disease) Patient self-management Physician decision support Physician wellness and resilience Social determinants of health
**Appraisal of family medicine AI-based tools**		
	Assessing and reviewing AI-based tools to ensure safe and effective integration and use in clinical practice.	Identification of potential AI adverse effects and potential solutions Quality improvement

^a^AI: artificial intelligence.

#### Element 4: Organization of AIFM-ed Curriculum Content

Family medicine postgraduate training is 24 months long in Canada. Given that the current curriculum is considered very heavy, educators and residents emphasized that the addition of another competency could be a burden to both educators and resident learners. They nonetheless agreed that AI curricula will eventually need to be added to that and an organized teaching structure would need to be established. Residents favored incorporating the teaching within the existing, already tight, 24-month core teaching, so that the benefits of longitudinal learning could be taken advantage of. The educators saw AI knowledge–based training during the first postgraduate year, followed by the development of AI-based clinical skills in the second postgraduate year. Educators proposed that if deeper AI education is needed, an additional third-year training program could be introduced for a select group of interested trainees to develop advanced AI skills in family medicine.

#### Element 5: Implementation of AIFM-ed Curriculum

Curriculum implementation will require the identification of appropriate resources (eg, educators and materials) along with educational strategies that will facilitate teaching activities and learner evaluation.

#### Curriculum Delivery

Residents highlight that AI education must be longitudinal, as it must be built upon throughout the medical education continuum. Furthermore, educators emphasized that residency is student-centered with learners coming from diverse backgrounds where they must replicate the actual tasks performed during in practice. Therefore, the learning theory of constructivism appears to be a sound and advantageous choice. This learning theory posits that learners actively construct their own learning by drawing upon their prior experiences [60].

There are several methods to implement an AI education curriculum to family medicine residents; however, there are certain methods that are recommended by both educators and residents. In terms of learning about the knowledge and background of AI (eg, review of AI concepts or the ethical, legal, and social considerations of AI), hybrid (web-based and in-person) courses with asynchronous web-based modules, and in-person workshops, problem-solving sessions could be applied. Residents emphasized that didactic large group lectures especially in regard to a novel topic such as AI would be less engaging. The learning of such content should be considered a refresher with emphasis on the context of AI in family medicine. Both educators and residents then suggest that the in-person sessions would serve as a space for questions and answers and problem-solving activities.

To execute these educational methods, human resources (eg, AI medical educators) and material resources (eg, existing AI-based tools) are pertinent. Educators and residents highlighted that experts in the field of AI and family medicine would be ideal; however, educators emphasized that the faculty challenges such as the current number of experts are limited to provide this education. To overcome this, residents suggested that once an AI curriculum is established, further educators could be sourced from recently graduated residents who completed the AI in family medicine curriculum. With respect to material resources such as family physician–focused AI-based tools, both groups emphasized that they must be validated before use in educational settings.

#### Assessment and Evaluation Methods

Residents emphasize that the assessment and evaluation methods for the curriculum should be simple in context and focus on learners’ participation and exposure. More specifically, learners should be able to have the capacity to demonstrate how to use AI-enabled tools and techniques in a health care setting. This can be seen through the completion of projects and problem-based and simulation-based assessments. Educators on the other hand emphasized taking into account Kirkpatrick’s 4 levels of training evaluation model [61], where assessments should be directly related to the activity’s learning objectives.

## Discussion

### The First Curriculum Framework for AI in Family Medicine (AIFM-ed)

In this study, we introduced a novel and evidence-based initial curriculum framework, that is, AIFM-ed developed for AI literacy education in family medicine postgraduate training. This systematically co-developed framework used a combination of validated methods including a comprehensive scoping review, resident and educator panel discussions, and the involvement of interdisciplinary experts in the field. During the development and cocreation of this framework, several key findings emerged. These include the crucial role of multiple resource partners and innovative practices when integrating AI educational content in family medicine education. For example, AI technology vendors specializing in health care, upcoming startups, and AI-focused organizations.

Furthermore, educators and residents stressed the importance of learning about the application of AI-based tools and simulating their use as a method of learning. Several innovative practices have already been implemented including case-based learning and flipped classroom models. Moreover, the adoption of AI-based tools can be diverse depending on its context (eg, teaching and learning and clinical practice) with several barriers and enablers. Additionally, the study identified several challenges in effectively integrating an AI curriculum framework into existing educational structures. These include the lack of AI definition standardization, the reduced urgency in practice due to the lack of time and resources, as well as the capacity to balance theoretical and practical curricular content.

### Interprofessional Collaboration and Resources

During the development of the AIFM-ed curriculum framework, several resource partners were identified when discussing the implementation of AI education in family medicine. Interprofessional collaboration within multidisciplinary teams is essential in order for an AI curriculum to be effective [62]. Other researchers emphasize this sentiment when listing their recommendations of ensuring a responsible integration of AI technologies in medical education [63]. This multidisciplinary team and resource partners may include several stakeholders such as nurses, social workers, epidemiologists, AI experts, data engineers, software developers, and patients [64]. Other resource partners identified included AI technology vendors specializing in health care, upcoming startups, and AI-focused organizations. Residents brought up the concept that AI-based tools and AI in general will substantially change in the future (eg, improved tools, systems, and integrations) and thus stressed the importance of continuous partnerships with other professionals in order for relevant information and AI tools.

Educators emphasized that they were unaware of many AI-based tools for patient support and were thus apprehensive in advocating for AI-based tools. Therefore, family physicians and other primary care team members (eg, administrative staff and nurses) should share AI-based tools and resources, when available and known, with other family physician and family medicine communities. Additionally, residents have suggested that before advocating or suggesting AI-based tools, a list of recommended AI-based tools must be developed and released from a medical organization such as the CFPC. Currently, there is a scoping review and inventory that has identified and evaluated published studies that have tested or implemented AI in primary care settings [20,65]. This can be a starting point for such a list of recommended AI-based tools.

### Application and Simulation of AI-Based Tools

Both educators and residents emphasize that a curriculum should focus on how to use AI-based tools (application) along with how to decide when to use and evaluate them (critical appraisal). Residents are already doing this comparatively as seen through their discussions of using ChatGPT, an AI-based chatbot launched by OpenAI that can be used as a digital consultant (eg, simple inquiries about diagnoses and treatment plans). One resident stressed that although they use ChatGPT at times for inquiries related to patient care, they are cautious of the information, as they are aware that ChatGPT can make mistakes and always consult other resources. As ChatGPT rises in prominence, its impact on medical education has been evident through the resident panel discussion and the literature [66,67].

The incorporation of AI content in medical education has already begun with innovative practices, which include case-based learning and flipped classroom models. Case-based learning incorporates real-world AI use cases, where AI is used in clinical practice as examples for physicians [68]. Through this learning approach, students have a better understanding of the technical aspects of AI, as it allows physicians to compare their thought processes with other students and critically reflect or challenge their assumptions and biases of AI and clinical practice [68]. One study assessed the capabilities of ChatGPT within the framework of a preclerkship case-based active learning curriculum. Although the AI chatbot is not comprehensive enough to serve as a textbook, it was shown to answer questions, generate test questions, and appropriately respond to prompts in case-based learning scenarios [69]. According to a scoping review of teaching AI ethics in medical education, 5 publications reported in using case-based learning when understanding ethical challenges [70]. Resident panelists believe that simulation of these tools is beneficial, as it allows residents to enjoy the learning process and realize how these AI-based tools would operate in actual clinical settings. During these simulation sessions or case-based learning approaches, educator panelists highlighted reviewing the capabilities and basic functions of AI-based tools.

Another practical example of incorporating AI content through innovative practices is the flipped classroom model approach. Flipped classroom models can consist of web-based content supplemented by in-person classroom sessions [71], a key observation reinforced by residents of the panel discussion. One study designed and evaluated a novel AI course for medical students using a flipped classroom model, and they found that attending the course can increase self-perceived AI readiness in medical students [71]. In addition, educators have also commented on facilitating AI learning by integrating family medicine AI-based tools in quality improvement projects, which has been emphasized and recommended by other researchers [72].

### Adopting AI in Education and Clinical Practice

Family physicians use AI, when implemented, primarily for diagnosis, detection, or surveillance purposes [20]. Although educators have flexibility in choosing from a wide range of AI tools, certain tools have proven to be particularly essential for effective integration. These include AI-enabled chatbots, clinical documentation support, and diagnostic decision support, which have shown to improve physicians’ efficiency and accuracy in their work [73-75]. However, there have been several barriers identified in previous reviews, which have made the adoption of AI-based tools difficult [76-78]. These issues include a lack of trust among educators, students, and clinicians; insufficient training and digital literacy; and resistance to change [77].

Additional challenges include data privacy and patient safety concerns, ethical and legal issues, interoperability issues, lack of funding, and inequities in access to AI tools—particularly between rural and urban settings [79]. In contrast, several strategies and enablers have been identified in order to better facilitate the adoption of AI and its continued use. These strategies include strategies fostering interdisciplinary collaboration between educators, clinicians, and AI developers; providing targeted training programs to build AI literacy; developing high-quality datasets for diverse use cases; and creating supportive regulatory frameworks [77]. Establishing national or local community networks to share resources and best practices, while leveraging trusted relationships within these networks, can also significantly enhance confidence in and adoption of AI-based tools. To identify relevant enablers and barriers to AI adoption of a certain audience, a comprehensive, stakeholder-centered approach is essential. For example, researchers in Canada conducted in-depth interviews with primary health care and digital health stakeholders and were able to ascertain their current barriers and potential facilitators of AI [80].

It is important to note that AI systems exist in diverse contexts and content with distinct implications, risks, and ethical and legal challenges depending on their application and domain. For example, in education, AI-enabled tools using large language models may offer personalized education, but biases may be propagated, inaccurate information may be generated, or students may overrely on AI, undermining their critical thinking skills [63,81]. In addition, there is potential for the exacerbation of inequities in accessing AI tools as well as the misuse of AI-generated content. In comparison, AI-enabled tools in clinical practice, such as decision-support systems, could carry risks of incorrect or biased recommendations that may directly impact patient outcomes [82,83], thus, raising ethical concerns about patient autonomy and safety as well as legal liability in cases of harm. Therefore, the differences of AI in each domain are important to understand in order to identify appropriate safeguards. Future research should conduct comparative analyses of AI’s risks, implications, and ethical and legal dimensions in educational versus clinical settings, examining factors such as accuracy, equity, accountability, and trust. These studies can inform best practices and policies to optimize AI’s potential while mitigating domain-specific risks.

### Curriculum Framework Challenges

During the development of this curriculum framework, there were several challenges in effectively integrating an AI curriculum framework into a family medicine residency training program. During the expert panel discussions, many experts emphasized the issue regarding the lack of standardization with the definition of AI. Although a definition of AI was chosen for the purpose of the panel, a specific and committed definition of AI within medical education has not been established [84-86]. Panelists argued that an AI definition must be properly explained to avoid confusion or misrepresentation. In relation to family medicine, a recent primer for AI in primary care was published, which provided the definition, “The field of AI is broad and rapidly expanding. The field is centred on how computers might be able to perform humanlike ‘intelligent tasks,’ such as summarizing large amounts of information or making inferences about a situation” [87]. The discussions regarding this framework highlight the necessity of a standardized AI definition for better development of teaching and learning content. This is especially true when specializing in different fields of medical education, including family medicine and primary care.

There is a need to introduce AI education within family medicine; however, the low urgency and priority to integrate this type of education at the moment were noted throughout the discussions. This can be due to the lack of AI-enabled tools for family physicians currently being developed, tested, and implemented in practice [88,89]. Furthermore, some residency programs lack the appropriate AI tools or are in lower-resource settings. As a result of the minimal exposure family physicians have with AI, their motivation to learn about the topic can also be reduced. This reduced priority of AI education competes with the CFPC’s 105 topics of family medicine curricula [4]. This is exacerbated by the fact that Canada is in a unique position, in which the length of residency training is only 2 years. In addition, the rapid advancement of AI introduces an extra layer of complexity. As new AI-based tools emerge and existing ones advance, educators and family physicians must frequently reassess and update their knowledge and skills. For example, the recent introduction of generative AI and generative AI tools such as ChatGPT has gained widespread popularity in medical and academic settings [90]. Thus, it is difficult to maintain a robust framework due to the inevitable rapid changes of AI in health care. Therefore, the eagerness to integrate this type of education within the curriculum should be met with caution to manage the expectations of both educators and learners.

A key observation made throughout the panel discussion was about the AI content and how much should a family physician know about AI. During the discussions, many of the participants voiced support on the application and appraisal of AI-enabled tools. This is especially challenging when residency is only 24 months, and there are no required AI educational programs presented in the Canadian undergraduate medical education system. Therefore, within the learning objectives, in regard to how much a family physician should know about AI remains undetermined. Further research must be conducted to investigate the level of AI education a family physician should be aware of. Overall, the aforementioned challenges must be addressed in order for this curriculum framework to be effectively implemented.

### Future Studies

Following the analysis, design, development, implementation, and evaluation model process, researchers may move forward to the implementation and evaluation of the AIFM-ed framework. During the implementation step, an educational program such as a course or workshop can be developed with the main concepts originating from the curriculum framework. The training for family medicine is already packed; thus, the implementation of this framework will depend on several factors including the current use of AI-enabled tools in family medicine training, previous training in AI (eg, the undergraduate foundation of AI), and the capacity of experienced teachers. However, once implemented, certain success indicators will need to be evaluated to understand its impact as well as any areas for improvement. Future studies could explore indicators such as the perceived impact of the framework, degree of implementation, as well as knowledge and skill apprehensions. These indicators can be evaluated through the framework-derived educational training program according to the Kirkpatrick model [61].

### Strengths and Limitations of This Study

This study had several strengths, including the formation of a national, multidisciplinary panel of family medicine educators. This diverse panel facilitated enriching discussions with varied expertise and insights, allowing for a comprehensive understanding of practical implications and current perspectives on AI education in family medicine postgraduate training. Additionally, by involving both educators and residents, the AIFM-ed curriculum framework ensures the representation from key stakeholders involved in the teaching and learning process of AI education. This co-design approach enhanced the relevance and applicability of the AIFM-ed curriculum framework. Regarding the overall development of this framework, a multi-method systematic approach was used, which includes a comprehensive systematic scoping review and multiple expert panel discussions. This approach allowed us to identify and build on existing AI curriculum topics and resources while also creating new ones. Furthermore, this structured and reproducible methodology ensures a robust foundation that can be used by other educators and researchers to develop training programs (eg, courses) following the established framework.

Despite the strengths, this study also had few limitations. First, the study was developed for programs in Canada, which limits its applicability to other countries due to the different medical education structures globally and their current relationships with AI. However, this could be a starting guide for other researchers to adapt it to their own context. Additionally, expert panel diversity was limiting, where the resident panel came from a single institution, which may further limit the generalizability of the framework. Furthermore, as the participants for the panel discussion were not randomized and were purposively recruited, the results may be subject to selection bias.

### Conclusions

We co-developed an AIFM-ed framework for family medicine residency training that outlines its curricular purpose, learning objectives, AI curricular topics, delivery methods, and evaluation strategies to be used by medical institutions. The AIFM-ed curriculum framework ultimately aims to enhance the education of future family physicians, equipping them to effectively integrate AI-enabled tools into their practice and patient care. It is hoped that this framework will provide further advocacy, productivity, and gradual change within the area of curriculum development and AI medical education. Overall, medical institutions are encouraged to begin equipping future physicians with the knowledge, skills, and confidence to effectively use AI-enabled tools, as these technologies will continue to grow within the field of health care and family medicine.

## Abbreviations

AI: artificial intelligence
AIFM-ed: Artificial Intelligence Training in Postgraduate Family Medicine Education
CanMEDS: Canadian Medical Education Directives for Specialists
CFPC: College of Family Physicians of Canada
PRISMA-ScR: Preferred Reporting Items for Systematic Reviews and Meta-Analyses extension for Scoping Reviews
SRQR: Standards for Reporting Qualitative Research
